# Comprehending Health of the Transgender Population in India Through Bibliometric Analysis

**DOI:** 10.3389/ijph.2024.1606598

**Published:** 2024-04-11

**Authors:** Papia Raj, Ashwani Dubey

**Affiliations:** Department of Humanities and Social Sciences, Indian Institute of Technology Patna, Patna, Bihar, India

**Keywords:** transgender, health, India, bibliometric analysis, literature review

## Abstract

**Objective:** In India research on health issues of transgender populations are very recent and limited though transgenders are an important sub-group of the population. Hence, this study attempts to understand the state of transgender health research in India through a systematic review of literature.

**Methods:** A systematic literature review was conducted using bibliometric analysis. Initially, 132 studies were identified, and only 37 articles meeting selection criteria were subsequently selected for review using PRISMA 2020 guidelines. The research landscape was examined with tools such as Biblioshiny, Arc-GIS (10.1), and Vos-Viewer.

**Results:** The review highlights that existing literature on transgender health in India mainly focuses on sexual health while neglecting their overall health status. It also emphasises the skewed geographical coverage of these studies. Based on the analysis, the interdisciplinary nature of the subject is illustrated in a three-field plot and through term co-occurrence. These indicate the need for culture-specific gender-affirmative services promoting a holistic approach to comprehend the health of transgender populations in India.

**Conclusion:** In India research on transgender health is lopsided and at an initial stage. There is a need to develop diverse research focus on various health issues of transgenders that should also be geographically representative. Future in-depth research on this subject will enable optimizing resource allocation, developing effective gender-inclusive policies, and support holistic planning for better health status of transgender people in India, and other countries with similar socio-cultural background.

## Introduction

Gender disparities in health are a global phenomenon although at varying degrees. The situations are more critical when we focus on gender minorities like transgenders, especially in culturally diversified countries like India. A succinct historical background would enable us to holistically contextualize the contemporary health status of transgender population in India. Search of academic literature reveals that the term Transgender was coined by John F. Oliven in 1965 [1]. According to authors [2], transgenders are individuals whose gender identity does not correspond with the sex assigned to them at birth. This includes persons identifying as trans men, trans women, or genderqueer. It also encompasses intersex individuals whose biological characteristics do not fall into the conventional male/female divisions. However, in the Indian context, various socio-cultural communities, including *Kinner, Hijra*, *Aravani, and Jogtha*, are encompassed within the broader spectrum of transgender identities [3]. Mention of these communities can be traced back to century-old texts, such as the *Vedas* and the *Puranas*, showcasing the inclusivity and recognition of their existence. These texts refer to the notion of *Tritiya prakriti* or *napunsaka*, which denotes the third gender. Similarly, Patanjali’s *Mahabhasya* posits that the Sanskrit grammar of three genders is derived from three natural genders, while the *Manu Smriti* emphasizes the biological basis of the three sexes. Even the Jain texts discuss in details about transgender people in India [4]. Thus, it can be contended that the transgender populations held a very important position in early Indian society and were an integral part of mainstream culture. However, during medieval period with various invasions in India, the culture and identity of transgenders suffered. These invaders were not able to comprehend the “third gender” based on their binary vision of genders; hence transgenders were allotted the role of gatekeepers of harems. This was followed by waves of colonization, where transgender individuals were categorized as criminal minorities and denied civil rights [5]. Especially the introduction of the Indian Penal Code (IPC/1861), commonly known as section 377 of the IPC, acted in 1861 and the Criminal Tribal Act of 1871-97 [4]. Thus, over a period of time, such discourses adversely affected the socio-economic status and wellbeing of transgenders. As a result, they encountered stigmatization and marginalization, resulting in restricted educational opportunities, diminished chances of employment, and an increasing susceptibility to violence and prejudice. These cumulatively contributed to adverse health conditions of this community [6, 7]. Nevertheless, such discriminatory views prevailed even after India gained independence until the landmark NALSA (National Legal Services Authority) judgement in 2014, which granted citizenship rights to transgender individuals [2]. Subsequently, the Indian Parliament enacted the Transgender Persons (Protection of Rights) Actin 2019 which aimed at ensuring equal rights and protection for the transgender population [8].

However, despite the significant progress represented by these legal measures in acknowledging transgender rights, obstacles still persist in achieving social acceptance and health equity for this community [9]. Also the absence of specific medical guidelines catering to the needs of transgender individuals in the country jeopardises the situation further. Thus, absence of affirmative healthcare initiatives exacerbates the persistent difficulties experienced by the transgender community [10]. Research suggests that transgenders often become victims of various mental health problems, such as chronic anxiety, thoughts of harming oneself, and feelings of hopelessness. These problems also harm their physical health and make them more vulnerable for contracting STDs and HIV [8]. Moreover, transgender people resort to self-medication primarily due to the unavailability of medical practitioners in the hospital and their lack of knowledge about transgender health [11]. Unfortunately, such self-medication practices carry severe, potentially life-threatening consequences, including kidney failure and cardiovascular problems. As a result, overall, transgenders face significant health challenges.

It needs to be acknowledged that like everyone else, transgender people have a right to good health. But as the preceding discussion shows, they encounter many obstacles. Apart from lack of free or inexpensive gender-affirming care, the absence of trans-friendly registration and admittance procedures and continued unfavourable experiences with the service providers also affect their health status [12]. Hence, these challenges emphasize the urgent need for comprehensive and inclusive measures to ensure fair healthcare access and improve the health and wellbeing of the transgender community. Therefore, this study aims to conduct a systematic literature review using bibliometric tools and knowledge network visualization, with the primary objective of identifying paucity of existing research on transgender health in India. Recognition of these gaps will enable formulation of gender-sensitive policies to ensure gender equality in health.

## Methods

**FIGURE 1:**
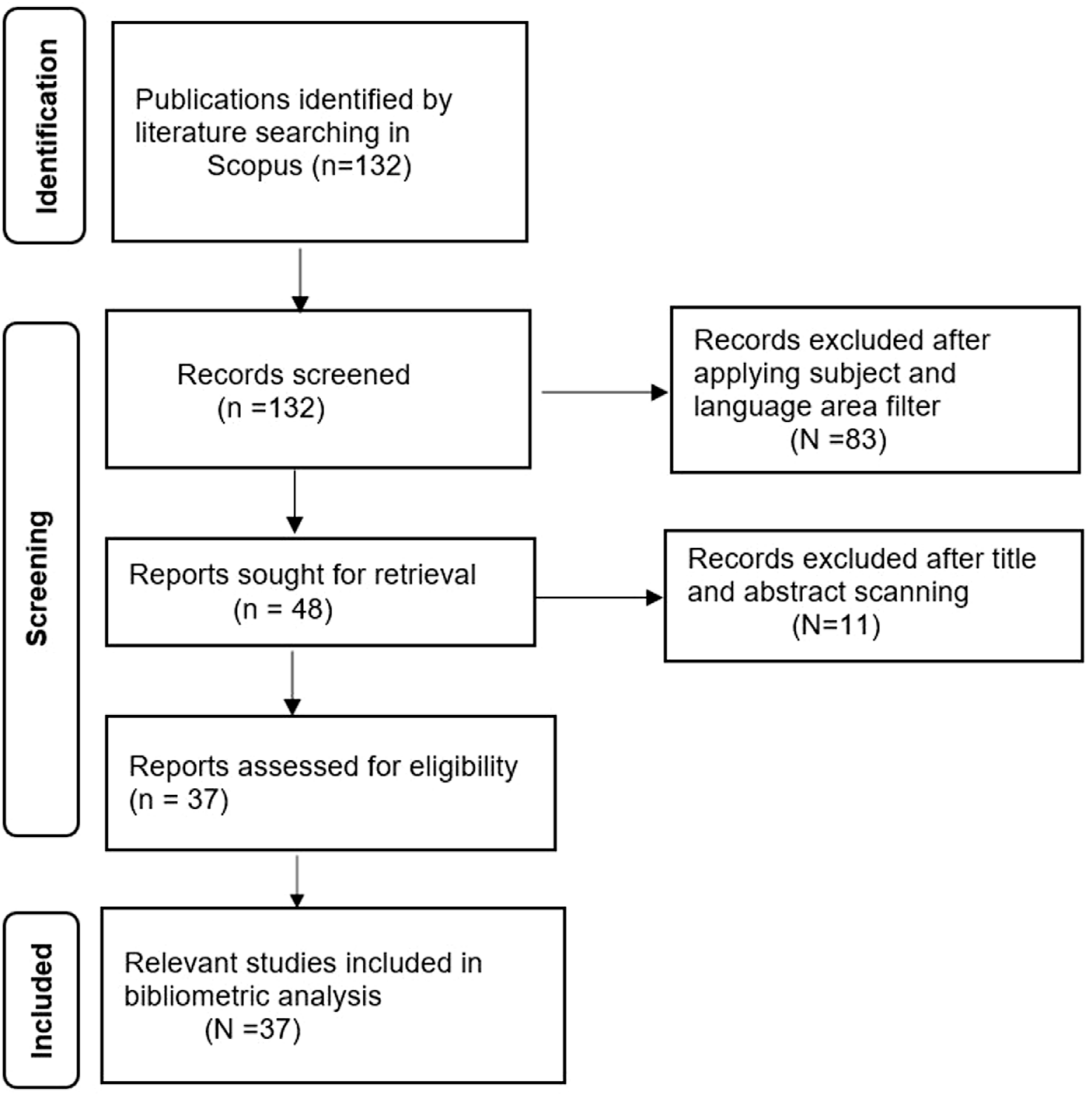
Selection of articles, adopted from Preferred Reporting Items for Systematic Reviews and Meta-Analysis 2020, Flow Diagram (India, 2022).

The landscape of transgender health research in India is examined using bibliometric analysis as a part of systematic review of literature. Literatures were selected through the Preferred Reporting Items for Systematic Reviews and Meta-analysis (PRISMA), 2020, as depicted in Figure 1 [13]. A thorough investigation of significant indexing databases, including Scopus, PubMed, and Web of Science, was conducted for bibliometric analysis. Then, Scopus was ultimately selected as the database for the literature search due to its large article collection and broad disciplinary coverage, which makes it possible to gather and evaluate a more comprehensive range of literature [14]. The keywords used in the search included “Transgender Health India,” “transgender health India,” and “Trans-gender health India” in the “Title, Abstract, and Keyword” sections of the database. The outcomes were consistent with all the keywords. Literatures were retrieved between the years 2011 and 2022 as before 2011 only one literature was available with the selected keywords and that too did not meet our inclusion criteria. It was also noted that most of the studies on transgender health were conducted from clinical and/epidemiological perspectives. Since gender is an important social determinant of health, through this review we wanted to develop an in-depth understanding of the research conducted on transgender health from perspectives of social sciences. Hence, this review was restricted to literatures published only within various disciplines of social sciences.

In the preliminary search 132 records were identified. These records were screened on the basis of two criteria-documents published in English; and only those published within disciplines of social sciences. Only 48 documents met these selection criteria. The second set of selection was based on the title and abstract of the documents and only 37 documents were eligible for the review. Various data processing and visualization techniques, including VOS-Viewer, Biblioshiny, ArcGIS (10.1), and Microsoft Excel are used to analyze the existing literature on transgender health in India [15]. Moreover, the globally most cited publication and three field plot of the collaboration map is created through Biblioshiny using R-Studio [16] while the spatial map is created with the help of ArcGIS (10.1) [17].

## Results

### Globally Most Cited Articles

Table 1 presents the most globally cited publications included in the study. It emphasizes that the article titled “Barriers to free antiretroviral treatment access among kothi-identified men who have sex with men and Aravanis (transgender women) in Chennai, India,” written by authors [10], received the highest total citations (*n* = 70) while author’s [18] article “Stigma, violence, and HIV vulnerability among transgender persons in sex work in Maharashtra, India,” received the most total citation per year (*n* = 8.86). This citation metric helps us to elucidate the fact that only a few of the published literature, which focuses on sexual health of transgender community, were cited several times. This reaffirms that until now, the major focus on transgender health in India is limited to sexual health. Thus, it indicates a lack of attention towards other health problems that need to be incorporated into academic discourse and are necessary for developing a holistic understanding of the health status of the transgender population.

**TABLE 1:** The five most cited documents (Generated by authors through Biblioshiny, India, 2022).

Year	Author	Title of paper	Journal title	Total citation	TC per year
2011	Chakrapani et al	Barriers to free antiretroviral treatment access among kothi-identified men who have sex with men and aravanis (transgender women) in Chennai, India	Psychological and Socio-medical Aspects of AIDS/HIV	70	5.38
2017	Ganju and Saggurti	Stigma, violence, and HIV Vulnerability among transgender persons in sex work in Maharashtra, India	Culture, Health & Sexuality	62	8.86
2012	Kalra	*Hijras*: the unique transgender culture of India	International Journal of Culture and Mental Health	38	3.17
2015	Horton et al	Contesting heteronormativity: the fight for lesbian, gay, bisexual and transgender recognition in India and Vietnam	Culture, Health, & Sexuality	21	2.33
2013	Kalra and Shah	The Cultural, Psychiatric, and Sexuality Aspects of Hijras in India	International Journal of Transgenderism	20	1.82

### Three-Factor Analysis

**FIGURE 2:**
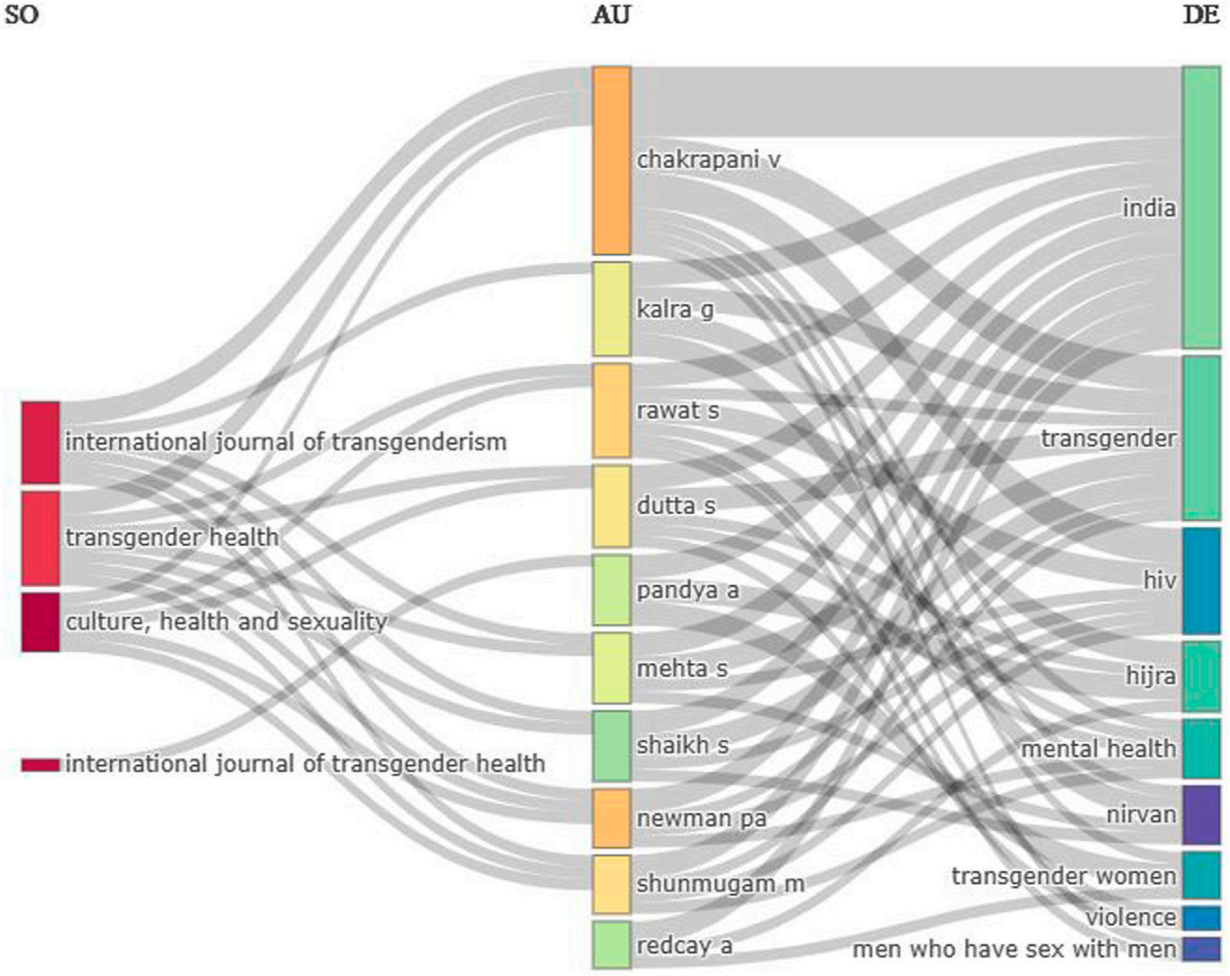
Three factor analysis (Generated by authors through Biblioshiny, India, 2022).

The three-factor analysis focuses on the collaborative nature of research and examines the extent of collaboration among authors, institutions, and publications. Figure 2 comprehensively depicts the research’s interdisciplinary collaborations among various journals, authors, and keywords. In this direction, the *International Journal of Transgenderism* collaborates with authors who specialize in different fields related to transgender health, including Venkatesan Chakrapani (sexual health researcher), Gurvinder Kalra (Consultant Psychiatrist), Sonal Mehta (from the Centre for Sexuality and Health Research and Policy), Simran Shaikh (member of the team in New India HIV/AIDS Alliance University of Mumbai), Murali Shunmugam (Manager-Programmes at the Centre for Sexuality and Health Research and Policy), and Peter A. Newman (Professor of Social Work, Canada Research Chair). These collaborations explore keywords such as “India,” “Transgender,” “HIV,” “Mental health,” “*Nirvana*,” “transgender women,” and “men who have sex with men.” This specifies a comprehensive investigation into topics related to transgender health, HIV prevention, mental wellbeing, and the experiences of marginalized communities.

Another significant collaboration is observed in the journal *Transgender Health*, involving authors Venkatesan Chakrapani, Shruta Rawat (Research Manager at The Humsafar Trust Mumbai, India), Sumit Dutta, Sonal Mehta, Simran Shaikh, Murali Shunmugam (researchers at the Centre for Sexuality and Health Research and Policy in Tamil Nadu, India), and Peter A. Newman. This collaboration explores keywords such as “India,” “transgender,” “HIV,” “*Hijra*,” “Mental health,” “transgender women,” “violence,” and “men who have sex with men.” It denotes a multidisciplinary approach to understanding various aspects of transgender health, including violence, mental health challenges, and the experiences of Hijra individuals.

Similarly, the *Journal of Culture, Health and Sexuality* collaborates with authors Venkatesan Chakrapani, Shruta Rawat, Sumit Dutta, Murali Shunmugam, and Peter A. Newman. Their research focuses on keywords such as “India,” “transgender,” “HIV,” “*Hijra*,” “Mental health,” “transgender women,” “violence,” and “men who have sex with men.” This collaboration highlights the intersectionality of culture, health, and sexuality within the context of transgender communities in India. Apurva Kumar Pandya (Director, Parul Institute of Public Health) collaborates with the *International Journal of Transgender Health*, investigating keywords related to “India,” “*Hijra*,” and “transgender women.” Additionally, author Alex Redcay (Associate Professor at Millersville University) explores the keywords “India,” “*Hijra*,” and “transgender women” in their research.

These collaborative networks and interdisciplinary research reflect a broad approach for studying and understanding transgender health, HIV prevention, and mental wellbeing of this marginalized community in India. The involvement of multiple disciplines, including psychology, social work, public health, and sociology, demonstrates a collective effort to advance knowledge and address the complex challenges faced by transgender individuals.

### Mapping the Geographical Distribution of Published Articles Across India

**FIGURE 3:**
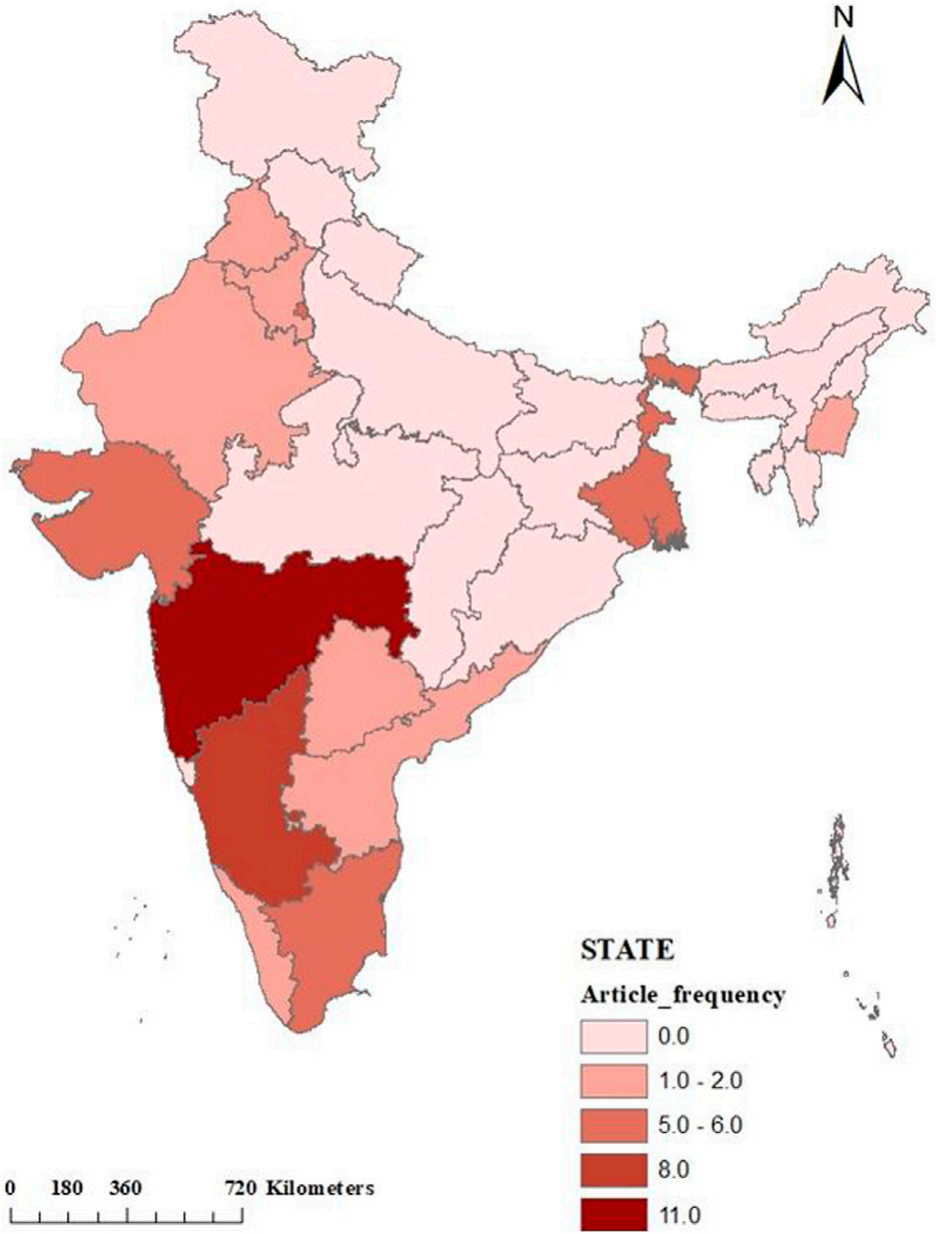
State-wise frequency of the articles (Generated by authors through ArcGIS, India, 2022).

A spatial analysis using ArcGIS (10.1) is shown in Figure 3, illustrates the geographical distribution of articles across India, thus identifying regions where no research has been conducted on transgender health. The selection of provinces for the spatial analysis is based on information provided in the articles considered in this study. It needs to be stated that if an article mentions multiple provinces as study areas, all of those provinces are included in the review. However, this spatial analysis does not consider articles that either do not mention the name of any specific province or cover the entire country. Interestingly, it is observed that research on transgender health issues is primarily concentrated in very specific regions and provinces. For example, Maharashtra demonstrates the highest research space, followed by Karnataka, West Bengal, Gujarat, Tamil Nadu, Delhi, Kerala, Telangana, Andhra, Punjab, Haryana, Rajasthan, and Manipur. No studies have been conducted in the provinces of Uttar Pradesh and Bihar, though they have highest number of transgender population according to Census of India, 2011. Even central India, including Madhya Pradesh, does not exhibit any studies on the transgender population. It is also evident from the map that the northeastern and northwestern regions of India have limited research representation, with only a few provinces being covered. Thus, it can be contended that the spatial distribution of research conducted on transgender population in India is very much skewed. This illustrates the pressing need to study health issues of transgender population across all provinces for a complete understanding.

### Term Co-Occurrence Analysis

**FIGURE 4:**
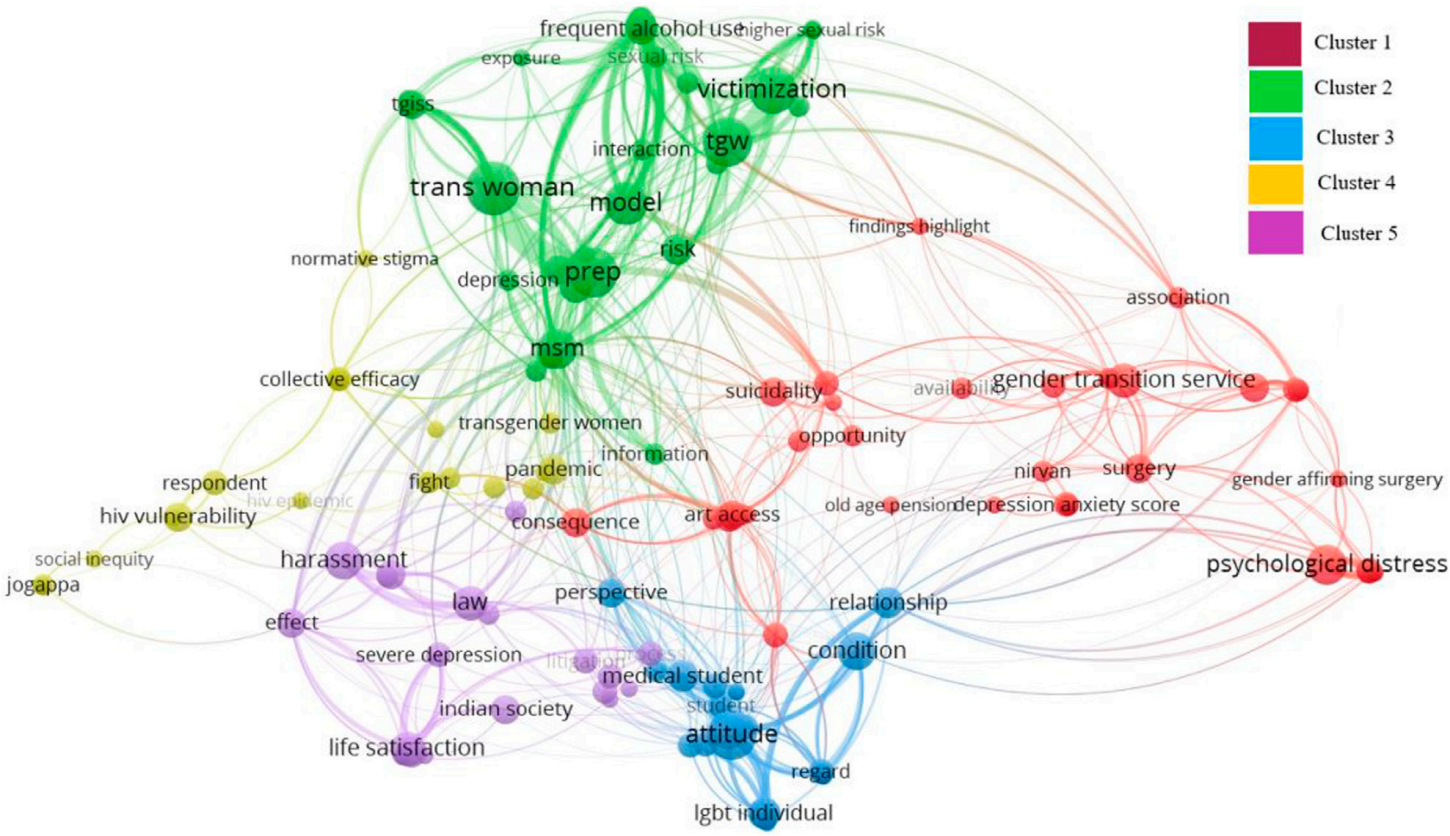
Term Co-occurrence (Generated by authors through VOS-viewer, India, 2022).

The term co-occurrence is used to examine the frequency with which words appear together in publications, indicating their thematic associations and connections. Of 1,268 identified co-occurrence terms, 329 met the requirement of appearing together at least twice. The Vos-viewer selected 60% most relevant words, resulting in 197 terms. After excluding 34 irrelevant terms, the analysis focused on 153 terms distributed across five clusters. Figure 4 uses colours to indicate clusters, representing groups of terms that share strong relationships. The clusters and nodes of research topics are depicted in Table 2. The following four themes are derived with the help of cluster’s nodes.

**TABLE 2:** The clusters and nodes of research topic (India, 2022).

Cluster	Color	Nodes (*n* = 153)
1	Red	Aravani, *Aravani* community, art access, association, availability, consequences, depression anxiety score, depressive symptoms, disclosure, dysphoria, employment, family rejection, female transgender person, finding highlights, gender-affirming surgery, gender diverse individuals, gender dysphoria, gender transition, gender transition services, healthcare provider, healthcare system, high prevalence, *hijras* transperson, HIV positive status, hormonal therapy, individual level, *Kothi,* national guideline, nirvana, old age pension, opportunity, physical violence, private hospital, psychological distress, public hospital, rape, reflection, social isolation, social strain, srs, suicidality, surgery, transgender congruence, transition
2	Green	Anxiety, assessment, causal epidemic, depression, discrimination experience, drug use, epidemic, evidence, exposure, frequent alcohol use, higher sexual risk, HIV risk, improvement, inconsistence condom use, information, interaction, low victimization, mental state, model, moderate victimization, MSM, mth, paf, pattern, prep, reliability, risk, sexual risk, syndemic, syndemic model, tgisq, tgiss, tgw, trans woman, twin heart, validity, victimization, violence victimisation, way interaction, willingness
3	Blue	Acute mental illness, attitude, awareness level, condition, cultural context, dental students, doctor, intellectual disability, LGBT, LGBT community, LGBT individual, LGBT person, medical personnel, medical student, mental health issue, MHL, minority stress, patient, perspective, regard, relationship, student, substance misuse
4	Yellow	Abuse, advocacy communication, collective efficacy, dimension, emergence, fight, *hijra* individual, HIV epidemic, HIV vulnerability, insight, interplay, *Jogappa*, legislation, marginalized community, media stigma, normative stigma, organisation, pandemic, respondent, social inequity, structural violence, struggle, transgender recognition, transgender women
5	Violet	Anxiety insomnia, decriminalization, effect, gender difference, general health questions, harassment, healthcare, homosexuality, Indian society, IPC, law, life satisfaction, litigation, moral policing, process, self-esteem, severe depression, tool

### Cultural Attributes

India is renowned for its rich cultural diversity, and within this cultural milieu, transgender communities like the *Hijra*, *Jogta*, *Jogappas*, and *Aravani* play distinctive and significant roles. Each community has specific cultural practices and beliefs that shape their identities. For example, the *Hijra* community has instrumental roles in religious ceremonies, for instance giving blessings during important life events, such as births and marriages [19]. The *Jogappas* community, also known as *Jogtas* or *Devadasis*, holds cultural significance in South India as the “brides of the deity” dedicated to serving temples through dance, music, and other religious rituals [20]. Similarly, the *Aravani* community often plays integral roles in both religious and social occasions [10]. These transgender communities encounter a range of challenges, including discrimination, limited access to healthcare, mental health issues, and higher rates of HIV/AIDS and other sexually transmitted infections. To address these difficulties, it is crucial to customize healthcare services to cater to the distinct needs these communities.

### Legal Considerations

The treatment of transgender populations as a societal pollutant during the colonial period and the introduction of legislation like section 377 of the Indian Penal Code, which criminalizes same-sex relationships, have profoundly impacted the legal and social landscape of queer subjectivity in India [11, 21]. The enduring consequences of this historical rejection and suppression are evident in the numerous disparities faced by transgender community in India, including limited access to healthcare, social stigma, discrimination, violence, harassment, poverty, economic marginalization, and limited educational and employment opportunities [20–23].

Transgender activists have been involved in challenging the gender binary approach and advocating for greater visibility and inclusion of transgender people [24]. Notably, the litigation for decriminalization of homosexuality in India has served as a mechanism for political and social change, contributing to increasing acceptance and recognition of the rights of transgender individuals [25]. However, structural factors such as family rejection, limited access to healthcare, social acceptance, and employment discrimination continue to hinder wellbeing of the transgender community [9]. Efforts to address these hurdles and promote the rights and wellbeing of transgender individuals in India including awareness campaigns, support programs, and policy reforms are needed [26].

### Social Attitude

The transgender community in India encounters significant difficulties due to the prevailing culture of prejudice and bias rooted in social factors. This societal outlook has led to substantial disparities in the physical and mental wellbeing of transgender individuals. They are susceptible to victimization, substance abuse, and frequent alcohol consumption, which contribute to the increased risk of HIV transmission within marginalized groups [27]. Research conducted by authors [28] emphasize the impact of stigma and discrimination on the health and wellbeing of both men who have sex with men (MSM) and transgender individuals. Similarly, authors [29] highlight the strong link between violence and negative mental health outcomes, such as depression, anxiety, and suicidal thoughts. It is also [30] revealed that transgender individuals experience lower life satisfaction, poorer mental health, and reduced self-esteem compared to males and females, primarily due to societal stigma. The pervasive social attitudes of stigma and discrimination against transgender individuals in India have significant implications for their overall health and wellbeing. It is crucial to gain a deeper understanding of their experiences through research to develop effective interventions addressing the stigma they face.

### Health Determinants

Transgender individuals in India face substantial health challenges including discrimination, limited awareness about transgender health concerns, and a lack of gender-affirming healthcare options [7, 31]. These barriers have resulted in higher rates of sexually transmitted infections (STIs) among transgender individuals [8], as well as difficulties in HIV/AIDS prevention and treatment [32]. Mental health issues such as depression, anxiety, and stress related to gender identity are also prevalent [5, 19, 33]. Additionally, transgender individuals face challenges in their gender transition journeys, including familial rejection and engagement in sex work, further marginalizing them [9]. The COVID-19 pandemic has exacerbated these challenges, with limited access to basic necessities and healthcare services [34, 35].

There is a need to prioritize mental wellbeing, reduce negative attitudes, and support the gender transitioning process [6, 9, 36, 37]. The education of medical students should be inclusive, creating an environment that promotes understanding and acceptance of transgender individuals in medical curriculum [38, 39]. Developing culturally sensitive and gender-affirming healthcare services is essential to improve access and outcomes for transgender individuals [29]. Gender-affirmative technologies (GATs) play a significant role in providing non-stigmatized services [40]. Further research is needed to understand gender-affirmative care and technologies to ensure their successful implementation in India, considering cultural nuances and promoting inclusivity.

## Discussion

The paper used bibliometric analysis which helped to evaluate the quantity and content of scholarly literature related to “transgender health in India.” An examination of the most globally cited articles indicates that sexual health has been the main thrust of research for transgender health in India. It emphasizes the dearth of other health-related studies. As a result, it is critical for academic research to widen its spectrum and include broader health dimensions, given that transgender people frequently confront many health difficulties that are not confined to sexual health. Correspondingly, mapping the geographical distribution of published articles across India reveals uneven spatial coverage of research on transgender health in India. The majority of study regions are concentrated in southern India, where NGOs are actively involved in research. Maharashtra emerges as the most prominent state, closely followed by other southern states in terms of transgender research. This necessitates evaluation of various health concerns prevailing among the transgender communities in all provinces, especially in Uttar Pradesh and Bihar, which have the largest number of transgender people in India [12]. The study highlights the importance of focusing on diverse aspects related to transgender health research. It also endeavours to concentrate on improving gender-sensitive healthcare and evaluating the implementation of legislation to improve standards of care to ensure the nation’s commitment to accomplishing the sustainable development target by 2030. These initiatives should be consistent with the goals set out in SDG 3 (Ensure healthy lives and promote wellbeing for everyone at all ages), SDG 5 (Gender Equality), and SDG 16 (Promote Peaceful and Inclusive Societies). Hence, based on this paper we contend that transgender health is one of India’s most priority areas of research to reduce overall gender disparity and promote gender equity.

### Limitation

The study is confined to a single Scopus database. This is because of the fact that both softwares used in the study- Vos-viewer and biblioshiny-can only use one database at a time. Other databases, such as PubMed, Google Scholar, Web of Science, and others might provide additional results that fall beyond the scope of this study. It also needs to be mentioned that data extraction from the Scopus database for this study was completed in December 2022, and any publications from 2022 onwards are not considered in this analysis.

## Author’s Note

Due to the evolving nature of search databases like Scopus, the method we employ to retrieve article information can vary over time. This can result in disparities between the archived dataset obtained and the data retrieved by directly using the search strategy within Scopus.
